# Heat shock protein 90 (HSP90) inhibitors in gastrointestinal cancer: where do we currently stand?—A systematic review

**DOI:** 10.1007/s00432-023-04689-z

**Published:** 2023-03-26

**Authors:** Christian Tibor Josef Magyar, Yogesh K. Vashist, Deborah Stroka, Corina Kim-Fuchs, Martin D. Berger, Vanessa M. Banz

**Affiliations:** 1 Department of Visceral Surgery and Medicine, Inselspital, Bern University Hospital, University of Bern, 3010 Bern, Switzerland; 2 Hamburg, Germany; 3 Department of Medical Oncology, Inselspital, Bern University Hospital, University of Bern, 3010 Bern, Switzerland

**Keywords:** Cancer, Therapeutic target, Heat shock protein (HSP), HSP inhibitors, HSP-based immunotherapy

## Abstract

**Purpose:**

Dysregulated expression of heat shock proteins (HSP) plays a fundamental role in tumor development and progression. Consequently, HSP90 may be an effective tumor target in oncology, including the treatment of gastrointestinal cancers.

**Methods:**

We carried out a systematic review of data extracted from clinicaltrials.gov and pubmed.gov, which included all studies available until January 1st, 2022. The published data was evaluated using primary and secondary endpoints, particularly with focus on overall survival, progression-free survival, and rate of stable disease.

**Results:**

Twenty trials used HSP90 inhibitors in GI cancers, ranging from phase I to III clinical trials. Most studies assessed HSP90 inhibitors as a second line treatment. Seventeen of the 20 studies were performed prior to 2015 and only few studies have results pending. Several studies were terminated prematurely, due to insufficient efficacy or toxicity. Thus far, the data suggests that HSP90 inhibitor NVP-AUY922 might improve outcome for colorectal cancer and gastrointestinal stromal tumors.

**Conclusion:**

It currently remains unclear which subgroup of patients might benefit from HSP90 inhibitors and at what time point these inhibitors may be beneficial. There are only few new or ongoing studies initiated during the last decade.

## Introduction

Heat shock proteins (HSP) are a family of molecular chaperones and key regulators of post-translational protein folding. They are known to be induced mainly by hyperthermia but can also be triggered by conditions such as ischemia, mechanical- or toxic stress (Chatterjee and Burns 2017). The first scientific publication on HSP was published in 1962 and since 1993 more than 1,000 publications are published annually (Ritossa 1962). A published guideline to classify HSP consists of five major families (HSPA, DNAJ, HSPB, HSPC, HSPD/E) (Kampinga et al. 2009).

Induction of HSP expression in response to stress is called heat shock response (HSR) and fulfills various tasks such as stabilizing protein folding and facilitating intracellular transport and signaling (Shevtsov et al. 2020). However, an increasing number of studies have shown that dysregulated expression of HSP plays a fundamental role in tumor development (Das et al. 2019; Liu et al. 2020; Shevtsov et al. 2020; Albakova and Mangasarova 2021). HSP overexpression can lead to tumor cell cytoprotection through suppression of apoptosis and is consequently classified as a client oncoprotein (Chatterjee and Burns 2017; Yang et al. 2021). Moreover, HSP appears to be involved in all hallmarks of cancer, such as mitosis, apoptosis, metastasis, angiogenesis and drug resistance (Hanahan and Weinberg 2000; Burrows et al. 2004; Calderwood et al. 2006; Chatterjee and Burns 2017; Calderwood 2018; Boroumand et al. 2018; Shevtsov et al. 2020; Duan et al. 2021; Albakova and Mangasarova 2021).

HSP90 is ubiquitously expressed in normal cells but is highly active in tumor tissue and its overexpression has been reported in various cancers as well as in infections, autoimmune-, cardiovascular- and cerebrovascular diseases (Moser et al. 2009; Garcia-Carbonero et al. 2013; Ghadban et al. 2016; Chatterjee and Burns 2017). HSP90 is a member of the HSPC family (Kampinga et al. 2009). HSP90 is involved in cancer-related signaling pathways such as HER-2, MET, BRAF, EGFR, STAT3, KRAS, PI3-K, c-Raf, p23, p53, FAK, TNFR-1 and Toll-like receptors (Burrows et al. 2004; Moser et al. 2009; Banz et al. 2009; Chatterjee and Burns 2017; Calderwood 2018; Boroumand et al. 2018; Kataria et al. 2019; Albakova and Mangasarova 2021). In 2003, Kamal et al. has shown that tumor cells from gastric and colon cancer cell lines have overexpressed and highly active HSP90 (Kamal et al. 2003). It has been suggested in several reviews, that HSP90 might be an effective target for the treatment of gastrointestinal (GI) cancers such as esophageal, gastric, hepatic, pancreatic, small intestine and colorectal cancer (Moser et al. 2009; Kim et al. 2009; Ghadban et al. 2016; Chatterjee and Burns 2017; Boroumand et al. 2018; Shevtsov et al. 2020; Duan et al. 2021). GI cancer represent ¼ of all newly diagnosed carcinomas annually worldwide (Arnold et al. 2020; World Health Organization 2020). Even though treatment options have significantly evolved in the past decades, cancer-related mortality remains significant, albeit differing according to the tumor origin (Arnold et al. 2019).

Several HSP90 inhibitors have been developed (Chatterjee and Burns 2017) (Table 1). The first generation of HSP inhibitors were derived from two potent natural inhibitors (Geldanamycin, Radicolol) with various derivatives developed thereafter (17-AAG, 17-DMAG, IPI-504 and WK88-1). The second generation of HSP90 inhibitors consist of synthetic radicolol-based derivatives (NVP-AUY922, AT13387, Ganetespib [STA-9090], GRP94). The STA-9090 binds to the N-terminal ATP-binding pocket of HSP90 interrupting the chaperone cycle (Ying et al. 2012). The new second generation of HSP inhibitors are purine and purine-like analogues designed to inhibit HSP90 using X-ray crystallography (CNF-2024, Debio 0932 [formerly CUDC-305, CUR-0374441], PU-H71). The development of HSP90 inhibitors classified as dihydroindazolone derivatives (SNX-5422) was abandoned early due to ocular toxicity (Rajan et al. 2011). A new group of selective cytosolic HSP90 inhibitors (Pimitespib (TAS-116)) has been developed with significant inhibition of tumor cell growth by blocking the NF-κB signaling pathway (Ikebe et al. 2022).

**Table 1:** Overview of available HSP90 inhibitors

Group		GI cancer publication
**1st generation**		
Natural		
Geldanamycin		
Radicolol		
Derivate		
17-AAG		Pedersen et al. (2015)
17-DMAG		
IPI-504		Wagner et al. (2013)
WK88-1		
**2nd generation**		
Synthetic Radicolol-based derivative		
NVP-AUY922		Subramaniam et al. (2015) Wainberg et al. (2013) Bendell et al. (2016) Chiang et al. (2016)
AT13387		
STA-9090	Ganetespib	Cardin et al. (2017) Cercek et al. (2014) Kwak et al. (2013) Goyal et al. (2013) Goyal et al. (2015) Goyal et al. (2020) Meehan et al. (2016) Thota et al. (2014)
GRP94		
Purine and purine-like analogue		
CNF-2024		
Debio 0932 [formerly CUDC-305, CUR-0374441]		
PU-H71		
Tropane-derived		
XL888		Akce et al. (2018)
**Others**		
Dihydroindazolone derivatives		
SNX-5422		Gutierrez et al. (2016)
Selective cytosolic HSP90 inhibitors		
TAS-116	Pimitespib	
Vaccine		
gp96 HSP-peptide complex	Vitespen	

At present, there are multiple ongoing clinical trials evaluating the efficacy of HSP90 inhibitors in GI cancers. In this study, we systematically reviewed the current outcomes of clinical trials in which HSP90 inhibitors were used to treat cancers of the GI tract which accounts for 35% of all global cancer-related deaths (Ferlay et al. 2018; Arnold et al. 2020b).

## Methods and materials

We screened the registry and results database for clinical trials (clinicaltrials.gov) as well as pubmed.gov for all data available online until January 1st 2022. The search term used in clinicaltrials.gov was ‘hsp’ (search engine automatically including term ‘heat shock protein’) and pubmed.gov for ‘(hsp) AND (cancer) Filters: Clinical Trial’.

Results were screened for the use of a HSP90 inhibitor in the selective setting of GI cancer. Pubmed.gov and google scholar were additionally searched for registered national clinical trial identifier (NCT) as extracted per clinicaltrials.gov. The selected studies were grouped and analyzed according to the primary tumor site. Exclusion criteria were trials including detection or diagnostic kits and trials without specific outcome data on GI cancer patients.

The status of the trials was documented as registered on clinicaltrials.gov. The primary endpoint was defined as overall survival (OS). Secondary endpoints were defined as progression-free survival (PFS), rate of stable disease (SD) and dose limiting toxicities. Reporting was done in accordance with the PRISMA guidelines (Page et al. 2021).

## Results

### Selection of studies

**Fig. 1:**
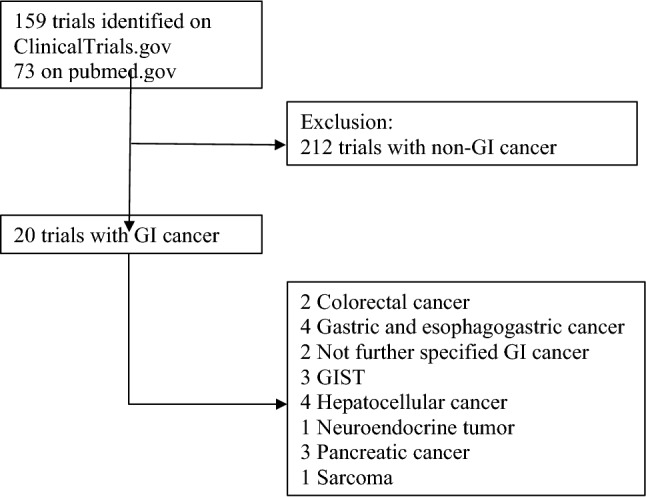
PRISMA study flowchart. *GI* gastrointestinal, *GIST* gastrointestinal stroma tumor

In total 159 studies including the search term HSP90 were identified on ClinicalTrials.gov, and 73 studies on pubmed.gov. Of the total 232 studies, 212 studies reported on non-GI related or non-solid tumors and were excluded (Fig. 1). All included studies (n = 20) are summarized in Table 2. Stratified by organ subgroup, the studies included colorectal (n = 2), gastric/esophagogastric (n = 4), not further specified gastrointestinal (n = 2), gastrointestinal stromal tumor (GIST) (n = 3), hepatocellular carcinoma (n = 4), neuroendocrine (n = 1), pancreatic (n = 3) carcinoma and sarcoma (n = 1). No studies were found for small intestinal or anal cancer. When dividing by era of publication: 1995–2000 (n = 1), 2001–2005 (n = 0), 2006–2010 (n = 6), 2011–2015 (n = 10), 2016–2020 (n = 2) and as per 2021 (n = 1).

**Table 2:** Overview of clinical trials of HSP90 in GI cancer

Study	Condition	Intervention	Study start	Status	Country	Phase	n =	NCT	Publication
Clinical and translational study of STA-9090	Colorectal cancer	STA-9090	2010	Completed	US	II	17	NCT01111838	Cercek et al. (2014)
Study of AUY922 and cetuximab in patients with KRAS wild-type metastatic colorectal cancer	Colorectal cancer	AUY922 and Cetuximab	2011	Completed	US	I	16	NCT01294826	Subramaniam et al. (2015)
STA-9090 in previously treated patients with advanced esophagogastric cancer	Esophagogastric cancer	STA-9090	2010	Completed	US	II	28	NCT01167114	Kwak et al. (2013) Goyal et al. (2020)
Phase II of AUY922 in second-line gastric cancer in combination with trastuzumab in HER2 positive patients	Gastric cancer	AUY922 and Trastuzumab	2011	Terminated	US, BE, FR, DE, IT, JA, KR, ES	II	21	NCT01402401	Wainberg et al. (2013)
PI3K inhibitor BYL719 in combination with the HSP90 inhibitor AUY922 in patients with advanced or metastatic gastric cancer	Gastric cancer	AUY922 and BYL719	2012	Completed	US, DE, JA, KR, CH, TW	I	18	NCT01613950	NDA/pending
Immunotherapy of gastric cancer with autologous tumor derived heat shock protein gp96	Gastric Cancer	gp96 vaccination	2014	Unknown	CN	I/II	45	NCT02317471	NDA/pending
Pembrolizumab and XL888 in patients with advanced gastrointestinal cancer	Gastrointestinal Cancer	XL888 and Pembrolizumab	2017	Active, not recruiting	US	I	50	NCT03095781	Akce et al. (2018)
Ganetespib and Ziv-aflibercept in refractory gastrointestinal carcinomas, non-squamous non-small cell lung carcinomas, urothelial carcinomas, and sarcomas	Gastrointestinal Cancer, other Neoplasms	Ziv-Aflibercept and STA-9090	2014	Terminated	US	I	5	NCT02192541	Meehan et al. (2016)
Study of Hsp90 inhibitor AUY922 for the treatment of patients with refractory gastrointestinal stromal tumor	GIST	AUY922	2011	Completed	US	II	25	NCT01404650	Bendell et al. (2016)
A study of AUY922 for GIST(gastrointestinal stromal tumor) patients	GIST	AUY922	2011	Unknown	TW	II	25	NCT01389583	Chiang et al. (2016)
Study evaluating IPI-504 in patients with gastrointestinal stromal tumors (GIST) following failure of at least imatinib and sunitinib	GIST	IPI-504	2008	Terminated	NDA	I	54	NCT00688766	Wagner et al. (2013)
STA-9090 in patients with advanced hepatocellular cancer	Hepatocellular Carcinoma	STA-9090	2010	Completed	US	I	16	NCT01665937	Goyal et al. (2013) Goyal et al. (2015)
Personalized cancer vaccine in egyptian cancer patients	Hepatocellular Carcinoma	Peptide cancer vaccine	2021	Recruiting	EG	I	10	NCT05059821	NDA/pending
GP96 heat shock protein-peptide complex vaccine in treating patients with liver cancer	Hepatocellular Carcinoma	gp96	2019	Not yet recruiting	CN	II/III	80	NCT04206254	NDA/pending
Immunotherapy of tumor with autologous tumor derived heat shock protein gp96	Hepatocellular Carcinoma	gp96 vaccination	2012	Unknown	CN	I/II	20	NCT02133079	NDA/pending
Safety and pharmacology of SNX-5422 plus everolimus in subjects with neuroendocrine tumors	Neuroendocrine Tumors	SNX-5422	2014	Completed	US	I	17	NCT02063958	Gutierrez et al. (2016)
Phase II study STA-9090 as second or third-line therapy for metastatic pancreas cancer	Pancreas Cancer	STA-9090	2010	Terminated	US	II	14	NCT01227018	Thota et al. (2014) Cardin et al. (2017)
Study of AUY922 in metastatic pancreatic cancer who are resistant to first line chemotherapy	Pancreas Cancer	AUY922	2012	Terminated	CA	II	15	NCT01484860	NDA/pending
Phase II trial of gemcitabine and tanespimycin (17AAG) in metastatic pancreatic cancer: a mayo clinic phase II Consortium study	Pancreas Cancer	17-AAG	2008	Completed	US	II	21	NDA	Pedersen et al. (2015)
Vaccine therapy in treating patients with recurrent soft tissue sarcoma	Sarcoma	Vitespen	1999	Completed	US	II	US	NCT00005628	NDA/pending

*BE* Belgium, *CA* Canada, *CH* Switzerland, *CN* China, *DE* Germany, *EG* Egypt, *FR* France, *GIST* gastrointestinal stromal tumor, *IT* Italy, *JA* Japan, *KR* Korea, *NDA* no data available, *TW* Taiwan, *US* United States

### Colorectal cancer

HSP90 inhibitors are currently being evaluated in two studies. The phase II study by Cercek et al. analyzed 17 patients with histologically confirmed at least first line chemotherapy-refractory metastatic colorectal cancer receiving treatment with intravenously administered STA-9090 until progression of disease, withdrawal of consent or unacceptable toxicity (NCT01111838) (Cercek et al. 2014). A median OS of 5.1 months (95% confidence interval [95% CI] 3.45–8.58 months) and a median PFS of 1.6 months (95% CI 1–2.8 months) was recorded. The authors concluded that STA-9090, as a single-agent HSP90 inhibitor, had no meaningful antitumor activity.

In a phase Ib trial from the US by Subramaniam et al., the combination therapy of AUY922 with cetuximab (EGFR-antibody) applied in 16 patients with at least second line chemotherapy-refractory metastatic colorectal cancer showed a median OS of 37.2 weeks (95% CI 4.9–115.1 weeks) and a median PFS of 7.9 weeks (95% CI 5.9–29.9 weeks) (NCT01294826) (Subramaniam et al. 2015). Patients demonstrating disease control (31.3%) had a median OS of 45.7 weeks (95% CI 37.6–115.1 weeks). The authors concluded that the administration of AUY922 was safe and that improved median survival was likely in part to be due to HSP90 inhibition.

### Gastric and esophagogastric cancer

Kwak et al. performed a phase II trial with STA-9090 in 28 patients with chemotherapy-refractory advanced esophagogastric cancer (NCT01167114) with data presented at the American Society of Clinical Oncology Annual Meeting in 2013 (Kwak et al. 2013) and published in 2020 (Goyal et al. 2020). The OS was 2.8 months. In one patient with a KRAS mutation in codon 12, a complete response was documented with treatment for 27.5 months. In two patients a tumor reduction of up to 20% was achieved. Due to insufficient evidence of STA-9090 activity, the trial was terminated prematurely. However, the authors concluded that there might be a subgroup of patients who might benefit from this treatment (Kwak et al. 2013; Goyal et al. 2020).

As part of a phase II study evaluating AUY922 for gastric cancer (NCT01402401), histological cell line analysis results were published, while there is still no data available for OS or PFS (Wainberg et al. 2013).

Results from one completed study evaluating AUY922 and BYL719 (NCT01613950) and one study with unknown status using gp96, also known as glucose-regulated protein, a secreted HSP90, vaccination (NCT02317471) are pending.

### Gastrointestinal stromal tumors (GIST)

Three studies were identified which specifically evaluated HSP90 inhibitors for GIST.

In 25 patients with chemotherapy-refractory GIST, the administration of AUY922 was evaluated (NCT01404650) (Bendell et al. 2016). The enrollment was stopped ahead of schedule due to slow accrual with a median OS of 8.5 months (95% CI 5.2–16.7 months) and mean PFS of 3.9 months (95% CI 2.5–5.3 months). SD was recorded for 60% of patients. Bendell et al. concluded that AUY922 in GIST could favorably improve PFS.

In 2013, the effect of IPI-504 in chemotherapy-refractory GIST and soft tissue sarcomas was analyzed in a phase I study (NCT00688766) (Wagner et al. 2013). Having enrolled 54 patients (37 GIST and 17 soft tissue sarcomas) a median PFS of 9.1 weeks (95% CI 6.7–12.0 weeks) was recorded (Wagner et al. 2013). In almost 50% of patients (n = 26) a progressive disease was documented, with two patients (4%) possibly succumbing due to treatment-related complications. In 6% (n = 3) adverse events such as increased pain or liver function test elevation were identified. GIST patients demonstrated a PFS of 10.6 weeks (95% CI 6.4–12.1 weeks) with SD in 73% at 6 weeks and SD in 16% of patients at 12 weeks (Wagner et al. 2013). Wagner et al. concluded that there is evidence of anti-tumoral activity of IPI-504 and that further studies are warranted (Wagner et al. 2013).

At the 2016 Gastrointestinal Cancer Symposium, data of a phase I study utilizing AUY922 for GIST with 25 patients (NCT01389583) was presented (Chiang et al. 2016). OS of 9.6 months (95% CI 0–21.1 months) with PFS of 2.4 months (95% CI 1.6–3.2 months) was found during a median follow-up time of 7.8 months (range 2.0–33.4 months). Modest antitumor activity was documented in these pretreated patients.

### Hepatocellular carcinoma

Four trials evaluating the treatment of HSP90 in hepatocellular carcinoma were identified.

A phase I study evaluated STA-9090 for advanced hepatocellular carcinoma (NCT01665937) with preliminary safety profile data published in 2013 (Goyal et al. 2013) and oncological endpoint data published in 2015 (Goyal et al. 2015). Out of 16 patients with chemotherapy-refractory advanced HCC, STA-9090 was administered in 14 patients. No data was available with regard to the previously administered chemotherapy. OS was 6.5 months (95% CI 3.60–12.27 months). Ten of the treated patients were evaluated for treatment response, with no patients achieving radiological signs of partial or complete response to treatment. One patient showed SD for 16 weeks. Median PFS was 1.8 months (95% CI 1.43–3.27 months) (Goyal et al. 2015). The authors concluded that an expansion to a phase II study was not warranted.

Results from three current clinical studies ranging from phase I to III for HSP90 in hepatocellular carcinoma are pending (NCT05059821, NCT04206254, NCT02133079).

### Neuroendocrine tumors

For the treatment of neuroendocrine tumors (NETs) the use of SNX-5422 was evaluated in an open-label and dose escalation study (NCT02063958) (Gutierrez et al. 2016). The enrolled patients had unresectable chemotherapy-refractory pulmonary or gastro-entero-pancreatic NETs. A total of 17 patients were enrolled. Fourteen patients were evaluated for efficacy, with SD in 57% and 14% with partial response. Gutierrez et al. concluded that further studies are needed to evaluate the efficacy of SNX-5422 use.

### Pancreatic cancer

The efficacy of STA-9090 in chemotherapy-refractory metastatic pancreatic cancer was evaluated in a phase II clinical trial (NCT01227018) with preliminary data presented in 2014 (Thota et al. 2014) and data published in 2017 (Cardin et al. 2017). Overall, 15 patients were enrolled, with one patient having active disease progression before treatment initiation. However, nine patients were excluded from the study due to radiological disease progression, two patients due to unacceptable toxicity, four patients withdrew for other reasons. OS was 4.57 months (95% CI 3.25–11.8 months) with PFS of 1.6 months (95% CI 1.15–4.7 months). There was 0% partial or complete response recorded. The trial was terminated due to lack of measurable efficacy.

In a phase II trial by Pedersen et al. the use of 17-AAG with gemcitabine (pyrimidine analogue) was evaluated in patients with metastatic pancreatic adenocarcinoma (Pedersen et al. 2015). Out of the 21 patients enrolled, 20 were available for analysis. Median OS was 5.4 months (95% CI 3.1–7.7 months) with a PFS of 2.6 months (95% CI 1.4–4.0 months) (Pedersen et al. 2015). Lack of treatment response and a 40% 6-month survival rate resulted in early termination of the trial (Pedersen et al. 2015).

### GI tract and sarcomas

Mehaan et al. performed a study with chemotherapy-refractory, progressive advanced carcinomas (including carcinomas of the not further specified GI tract, lung, urothelial lining and sarcomas) (NCT02192541). Having enrolled five patients which were treated with STA-9090 and ziv-aflibercept (vEGFR-antibody), they terminated the study early, due to findings that suggested an elevated toxicity of this combination (Meehan et al. 2016).

The phase Ib trial with XL888 with pembrolizumab (PD-1-antibody) in chemotherapy-refractory stage IV or locally advanced unresectable GI (pancreatic and colorectal) adenocarcinoma is currently still recruiting patients (NCT03095781) (Akce et al. 2018).

## Discussion

To our knowledge this is the first systematic review of clinical studies evaluating HSP90 inhibitors in GI cancers. Previous studies and reviews have focused on the pathophysiological or biochemical aspects of HSP inhibition (Hanahan and Weinberg 2000; Burrows et al. 2004; Calderwood et al. 2006; Moser et al. 2009; Banz et al. 2009; Neckers and Workman 2012; Garcia-Carbonero et al. 2013; Ghadban et al. 2016; Chatterjee and Burns 2017; Calderwood 2018; Boroumand et al. 2018; Das et al. 2019; Shevtsov et al. 2019, 2020; Liu et al. 2020; Costa et al. 2020; Boliukh et al. 2021; Albakova et al. 2021; Duan et al. 2021; Yang et al. 2021; Albakova and Mangasarova 2021). Multiple phase I and II trials using HSP90 inhibitors for GI tumors have been conducted or are still ongoing. Thus far, no specific GI cancer or patient subgroup has been identified, which might benefit from HSP90 inhibition. The number of new and ongoing studies has dropped during the last decade (Table 2).

It must be emphasized, that most patients included in the studies are refractory to conventional cancer therapy or show poor response, resulting in a selection bias. Currently, treatment of colorectal cancer with the second generation radicolol-derived inhibitor AUY922, and GIST treatment with AUY922 or IPI-504 in and SNX-5422 in NET, based on clinical phase I and II trials, supports HSP90 as a possible target. Garcia-Carbonero et al. hypothesized promising activity in certain cancer subgroups in a narrative review of HSP90 (Garcia-Carbonero et al. 2013). Boroumand et al., as well as Moser et al., both narratively reviewed pre-clinical data, suggesting the possibility of implications for HSP90 inhibitors in cancers of the GI tract (Moser et al. 2009; Boroumand et al. 2018). Data on combination treatment with immune checkpoint inhibitors (ICI) is sparse, nevertheless it has been hypothesized, that a treatment combination of HSP90 with ICI could be of interest.

The three malignant neoplasms of the GI tract with the highest rate of mortality in Europe are colorectal, gastric and pancreatic cancer (2018). HSP90 inhibitors can target several molecular targets implicated in the development of these cancers.

In colorectal cancer KRAS (including Raf/Ras/MEK/Erk), EGFR, c-Met, Apo2L, FAK, NF-κB are plausible molecule targets (Moser et al. 2009; Therkildsen et al. 2014; Alwers et al. 2019; Henderson et al. 2019; Reynolds et al. 2019; Levin-Sparenberg et al. 2020; Afolabi et al. 2022). HSP90 inhibition of HIF-1α with STA-9090 (Cercek et al. 2014), and inhibition of c-Met and FAK using AUY-922 appear to demonstrate a clinical activity and disease control (Subramaniam et al. 2015).

In gastric cancer HER-2, EGFR, Akt, HIF-1α and other proteins—all linked to HSP90—appear to be potential targets (Iacopetta et al. 1999; Scartozzi et al. 2004; Gravalos and Jimeno 2008; Nobili et al. 2011). Clinical data however shows no benefit of blocking the HIF-1α pathway using STA-9090. Gastric and esophagogastric cancer treatment options are currently under debate and guidelines differ internationally and intercontinentally (Moehler et al. 2015).

In pancreatic cancer, IGF-IR, STAT3, HIF-1α, IL-6, IGF-1, amongst others, have been described to be active and linked to the HSP90 pathways (Bruns et al. 2000; Wei et al. 2003; Xu et al. 2005; Lang et al. 2007). Clinical trials currently show a lack of response using STA-9090 and 17-AAG in combination with gemcitabine (Pedersen et al. 2015; Cardin et al. 2017). Pancreatic cancer has a poor prognosis with a 5-year survival rate of < 20% (Ghadban et al. 2017). Recent studies have demonstrated that > 80% of surgical resections have a microscopically positive resection margin, possibly being a relevant confounder in part the poor survival of patients with pancreatic cancer, even after radical resection (Butturini et al. 2008; Holm and Verbeke 2022). With this knowledge potent adjuvant chemotherapy may be the most important aspect in successful treatment (Ghadban et al. 2017). Currently, the chemotherapeutic options are very limited with the most commonly used treatment regimens being gemcitabine ± capecitabine in the adjuvant (Neoptolemos et al. 2017) and gemcitabine ± nab-paclitaxel in the palliative setting (Von Hoff et al. 2013), whereas 5-fluorouracil, irinotecan and oxaliplatin are used for both adjuvant (Conroy et al. 2018) and palliative treatment (Conroy et al. 2011). However, survival rates remain low for metastatic disease, with most patients not surviving longer than 12 months (Von Hoff et al. 2013).

Interestingly, while efficacy of HSP90 inhibitors in GI cancer still needs to be established, HSP90 inhibition seems to be efficient for other cancers such as breast cancer with HER2-amplification. In patients with metastatic breast cancer there is a median OS 17 months (95% CI 16–28 months) or clinical benefit rate (complete response, partial response or stable disease) of 59% (Modi et al. 2007, 2011; Kong et al. 2016). In patients with non-small-cell lung cancer an response rate of 7–32% was found (Sequist et al. 2010; Garcia-Carbonero et al. 2013; Felip et al. 2018).

Several developments may improve the use of HSP90 inhibitors for cancer therapy. For example, further characterization of the molecular pathways influenced by HSP90 inhibition may improve its targeted use. Pharmacological modifications, such as improving water-solubility would reduce the barrier for using of a number of HSP90 inhibitors in a clinical setting (e.g. IPI-493, KW-2478, MPC-3100 etc.). Finding an easy way to deliver HSP90 inhibitors is crucial for its acceptance in human clinical trials (Ghadban et al. 2016). These developments may open new trials for patients with GI cancers that may benefit from HSP90 inhibitors.

## Conclusion

At present, it remains unclear which subgroup of patients with GI cancer might benefit from HSP90 inhibitors in addition to classical treatment regimes. Further verification in phase II and III as well as larger scaled clinical trials are mandatory if use of HSP90 inhibitors is to be established in a clinical setting.

## Data Availability

Data can be provided upon request.

## Declarations

### Registration information

This review was not registered.
